# Retroperitoneal ectopic pregnancy: successful expectant management in condition of early pregnancy failure

**DOI:** 10.1186/s12884-023-05909-7

**Published:** 2023-08-22

**Authors:** Diep Ngoc Le, Phuc Nhon Nguyen, Phuong Hai Huynh

**Affiliations:** 1 Department of Laparoscopy, Tu Du Hospital, Ho Chi Minh city, Vietnam; 2 Department of High-risk Pregnancy, Tu Du Hospital, 284 Cong Quynh, Pham Ngu Lao ward, District 1, Ho Chi Minh city, 730000 Vietnam; 3 Tu Du Clinical Research Unit (TD-CRU), Tu Du Hospital, Ho Chi Minh city, Vietnam; 4 Faculty of Imaging Diagnosis, University of Medicine and Pharmacy HCMC (UMP), Ho Chi Minh City, Vietnam

**Keywords:** β-hCG, Ectopic pregnancy, Expectant management, Early pregnancy failure, Retroperitoneal ectopic pregnancy, Ultrasound

## Abstract

**Background:**

Retroperitoneal ectopic pregnancy (REP) refers to abnormal implantation of the fertilized egg in the retroperitoneal cavity. REP can be divided into pelvic and abdominal positions. Extremely rare, the incidence of REP is less than 1% of ectopic pregnancy (EP). Herein, we report the first case of paraaortic-located REP in association with successful expectant management, thus raising awareness among healthcare providers, particularly in low-resource settings.

**Case presentation:**

A reproductive-age woman presented at our tertiary referral hospital because of amenorrhea and a positive pregnancy test. Based on serial serum β-hCG levels and imaging modalities including transabdominal ultrasound, transvaginal sonography, and magnetic resonance imaging (MRI), a REP of 7–9 weeks of gestational age adherent to abdominal paraaortic region was detected. Since the pregnancy was spontaneously arrested without clinical symptoms, expectant management was first indicated following careful evaluation. After a 1-month follow-up, the ectopic mass naturally degenerated without complications and her β-hCG concentration returned to a negative value. Therefore, the patient recovered completely and avoided unnecessary surgery as well as toxicity of medical treatment when using systemic methotrexate.

**Conclusions:**

In addition to transvaginal and transabdominal ultrasound, MRI is necessary for the diagnosis of nonviable REP. Alongside the great vessels in the abdominal cavity should be taken into consideration in all suspected cases relating to this rare entity. Expectant management may be carefully indicated in conditions of nonviable REP and unruptured REP, where applicable.

## Highlights

Accompanying transvaginal ultrasound and transabdominal ultrasonic scan and MRI are necessary for a high index of suspicion of REP.

Alongside the great vessels in abdominal cavity should not be neglected in suspected case related to this rare entity.

Expectant management may be carefully indicated in conditions of absence of fetal cardiac activity and nonruptured REP, where applicable.

## Introduction

Retroperitoneal ectopic pregnancy (REP) refers to the implantation of a fertilized egg in the retroperitoneal cavity. REP can be divided into pelvic and abdominal positions [1]. The location is related to many different structures such as the kidneys, pancreas, abdominal para-aortic region, iliac artery, and obturator fossa [2]. Along with the development of assisted reproductive technology in the past decades, the number of ectopic pregnancies has increased, including at uncommon sites [3–5]. Among them, REP is exceedingly rare, accounting for less than 1% in ectopic pregnancies. Before 2021, 25 cases were reported in the literature following the report of Wen et al. [6]. Until today, a total of 36 cases have been found in the PubMed database [7, 8]. This uncommon site increases the maternal mortality rate higher than other sites in the first trimester. The mortality rate is 5.1 per 1000 cases [6]. However, the development mechanism of REP remains unclear [9]. Risk factors include reduced or impaired tubal transport activity, increased tubal receptivity for blastocyst implantation, tubal damage due to surgery or infection, peritoneal defects, and in vitro fertilization (IVF) [1].

Regarding clinical characteristics, symptoms can range from asymptomatic to severe manifestation associated with a ruptured EP which leads to immediate hypovolemic shock and death [1, 10]. Similar to almost all ectopic pregnancies, the common symptoms of REP include amenorrhea, abdominal pain, and vaginal bleeding [7]. Early diagnosis is usually difficult because of extremely specific location of the REP mass [11]. An accurate diagnosis is often made following the exclusion of other common sites. Transabdominal sonography (TAS) and transvaginal sonography (TVS) are first-line tools for assessment of REP [12]. However, some early ectopic pregnancies require additional imaging modalities such as MRI and computed tomography (CT) scans due to missed diagnosis on ultrasound [1, 13, 14].

Treatment includes surgical intervention, medical treatment with methotrexate, and expectant management [6]. In some cases, after failed medical treatment, a surgical method is indicated with the cooperation of an experienced multidisciplinary team [3]. Recently, Lorenzo et al. have mentioned a case of REP requiring of surgery after a failure of methotrexate treatment [15]. Moreover, laparotomy must be the preferred method of treatment in ruptured REP cases with severe hemorrhage [10].

Herein, we hereby describe an uncommon case of REP at our tertiary referral hospital and review the literature (Table 1). To our knowledge, this is the first REP case of paraaortic location with successful expectant management in the literature, with neither medical treatment nor surgical removal. Through this report, we aimed to increase physician awareness regarding REP in abdominal ectopic pregnancies and suggest an option management in condition of early failure of pregnancy.

**Table 1:** Retroperitoneal ectopic pregnancy in the last 5 years

Authors/ year	Gravida, parity Maternal age and Gestatioal age	Symptoms	Risk factors	Localization and size mass on imaging scan	β-hCG (mUI/ml)	Management	Outcomes
Salma et al. (2017) [19]	-35 yo -G4P2 − 7wk amenorrhea	asymptomatic	None	a large mass in the left para-aortic region, consisted of a GS with an embryo with positive cardiac activity. -the diagnosis was confirmed by MRI.	rose from 29,386 to 60,000	laparotomy due to lack of laparoscopic equipment	- β-hCG declined from 731 mIU/ml at day 1 to 55 mIU/ml at day 7 post surgery. -The patient left on the 7th day.
Yang et al. (2017) [2]	-32 yo -G5P1 -amenorrhea for 38 days	-lower abdominal pain, -rectal tenderness	-CS -pelvic infection and/or inflammation -circular peritoneal defect	-location at lateral to the left sacrocervical ligament, anterior to the left ovarian fossa, and next to the lower edge of the left broad ligament. -21 × 14 × 20 mm.	1880	laparoscopy	-EBL was at 300 ml -β-hCG returned to negative value after 29 days
Yang et al. (2018) [13]	-34 yo -G2P0 − 52 days of amnenorrhea	-a bellyache radiating to the right waist - dizziness, flustered, fatigue, thirsty, and urinary incontinence.	none	CT examination suggested that it was “retroperitoneal hemorrhage” in the right paraaortic region below the right kidney.	6803	laparotomy	recovery
Velemínský et al. (2018) [22]	-38 yo − 7 wks	-no clinical symptoms -admission for missed abortion/anembryonic pregnancy	none	a 27 mm GS with yolk sac and 13 mm embryo without any heart pulsation above the vena cava inferior was identified.	33,742	laparotomy after laparoscopic confirmation	discharged on the 9th day
Park et al. (2018) [10]	-30 yo -G4P3 -8 wks 6 d	hypovolemic shock with an acute abdomen.	none	retroperitoneal hematoma at the level of the kidneys	40,532	laparotomy for 2 times	-EBL at > 2.5 L - The patient received a total of 7 units of packed RBCs, 8 units of FFP, and 3 units of cryoprecipitate. -discharge on postoperative day 10 and -β-hCG returned to normal limit after 4 wks.
Zhang et al. (2018) [12]	-29 yo − 2 months of amenorrhea	left lower flank pain for 10 days	none	-close to the left side of the abdominal aorta. − 41 × 29 mm - visible yolk sac and an embryo	rose from 16,453 to 36 312	laparotomy	Not mentioned.
Lu et al. (2019) [23]	-31 yo -G2P1 − 54 days of amenorrhea	-spotting -lower abdominal pain for 8 days	-right salpingectomy for EP	-TAS revealed a GS 3.0 × 2.3 cm, with yolk sac and fetal cardiac activity, located adjacent to abdominal aorta and inferior vena cava.	47,440	laparoscopy	recovery
Huang et al. (2019) [20]	Case 1: -37 yo -G4P1 - amenorrhea for 65 days	asymptomatic	-CS -bilateral salpingectomy -embryo transplantation of 2 cryopreserved embryos.	-color Doppler US showed a GS (4.2 × 4.2 cm) in the lower pole of the left kidney. - The fetal heart was visible, and the sac was in close proximity to the abdominal aorta.	rose from 88, 165 to 92, 079	Computed tomographic-guided methotrexate injection in the gestational sac	recovery
	Case 2: -31 yo -G2P0 - amenorrhea for 65 days	asymptomatic	Laparoscopy for ectopic tubal pregnancy	CT revealed the presence of a GS with an embryonic shadow (3.4 × 2.9 × 4.6 cm) located in front of vertebra L3 and in between the abdominal aorta and inferior vena cava	97, 333		-recovery in good condition - β-hCG normalized at 100 days
Le et al. (2020) [16]	-31 yo -nulliparous -6 wks	acute epigastric pains, tenderness of the left flank area, no vaginal bleeding	-bilateral salpingectomy -IVF-ET	-left abdominal para-aortic region -5 mm -no embryo	20,625	laparotomy with a multidisciplinary team	discharged on the 4th postoperative day
Hou et al. (2021) [24]	-29 yo -G2P1 - amenorrhea for 48 days	acute left abdomen	not mentioned	-location at abdominal aorta and left common iliac artery -27 × 25 × 20 mm	28,746	-mifepristone 50 mg three times a day for 3 days. -laparotomy due to hematoma.	-EBL was 1100 mL, and 800 mL of RBCs and 400 mL of FFP were transfused. -recovery well after 2 months.
Wen et al. (2021) [6]	-28 yo -G4P2 -amenorrhea for 60 days	soreness of the left lower quadrant of the abdomen and amenorrhea	CS	4 × 3 cm mass in front of the middle and upper poles of the left kidney.	99,286	-laparoscopy and local methotrexate (50 mg/m^2^)	discharged on the 3rd day
Nguyen et al. (2022) [17]	-34 yo -G3P2 -31 days after ET	mild vaginal bleeding	-a history of bilateral salpingectomies due to 2 previous tubal pregnancies -IVF	-location next to the right common iliac artery -20 × 25 mm and featured a visible yolk sac.	29,242	-twice laparoscopy and then, laparotomy	β-hCG returned to normal limit after 4 wks.
Xu et al. (2022) [7]	− 29 yo -nulliparous -50-day amenorrhea	upper abdominal pain	none	4.5 × 4.0 × 3.0 cm, tightly adherent to the surface of inferior vena cava and the left side of abdominal aorta.	65,004	-systemic methotrexate. -potassium chloride solution injection into the gestational sac. -laparotomy	-recovered uneventfully -β-hCG returned to normal range on the 23th postoperative day.
Yuan et al. (2022) [18]	-32 yo -G2P0 − 40 days after IVF-ET	none	-right salpingectomy - IVF-ET	MRI showed that 1 oval signal measuring approximately 30 × 28 × 35 mm was detected at the gap between the aorta anterior to the third lumbar vertebra and inferior vena cava.	not mentioned	laparoscopy	recovery
Lorenzo et al. (2022) [15]	-33 yo -nullipara − 8 wks	acute abdominal pain	none	a live fetus in the left posterior parametrium on US -about 3 cm in size	820	laparoscopy and MTX	-discharge on 3rd day -β-hCG became negative in 20 days.
Ren et al. (2022) [14]	-30 yo -G5P2 − 47th day after the last menstrual period.	asymptomatic	-2 CS -1 medical abortion	an abdominal computed tomography (CT) demonstrated a 2.2-cm GS located on the surface of the inferior vena cava near the fourth lumbar vertebrae - a fetal pole and a heartbeat on US	rose from 11,141 to 17 351		
Our case	-38 yo -G5P2 -7-9 wks	asymptomatic	-CS -salpingectomy for EP	-adherent to the inferior vena cava, the abdominal aorta -4.6 × 5.6 × 5.4 cm	51,586	expectant management without further intervention	-β-hCG returned to negative after 1 month discharge

*CT: computed tomography, CS: cesarean section, D: days, EBL: estimated blood loss, EP: ectopic pregnancy, MTX: methotrexate, MRI: magnetic resonance imaging, GA: gestational age, GS: gestational sac, GP: gravida and parturition, IVF-ET: in vitro fertilization/embryo transfer, FFP: fresh frozen plasma, RBCs: red blood cells, US: ultrasound, yo:years old, wks:weeks*

## Presentation case

A 38-year-old female patient (G5P2) was transferred to our hospital owing to suspicion of abdominal ectopic pregnancy. Her obstetric anamnesis included one vaginal birth, one cesarean delivery, one ectopic pregnancy with salpingectomy, and one preterm birth at 22 weeks GA. She had also undergone an appendectomy in the past year.

On admission, the patient was stable. A general physical examination result was unremarkable. On gynecological examination, the uterus and bilateral ovaries were normal in size. The patient was asymptomatic and denied the use of urgent contraceptive pills. She complained of a retarded menstrual cycle of two weeks and her urine human chorionic gonadotropin (HCG) test was positive. Laboratory tests revealed the positive pregnancy tests. The quantitative serum beta-human chorionic gonadotropin (β-hCG) titer test was 51,586 m-international units per milliliter (mIU/mL).

**Fig. 1:**
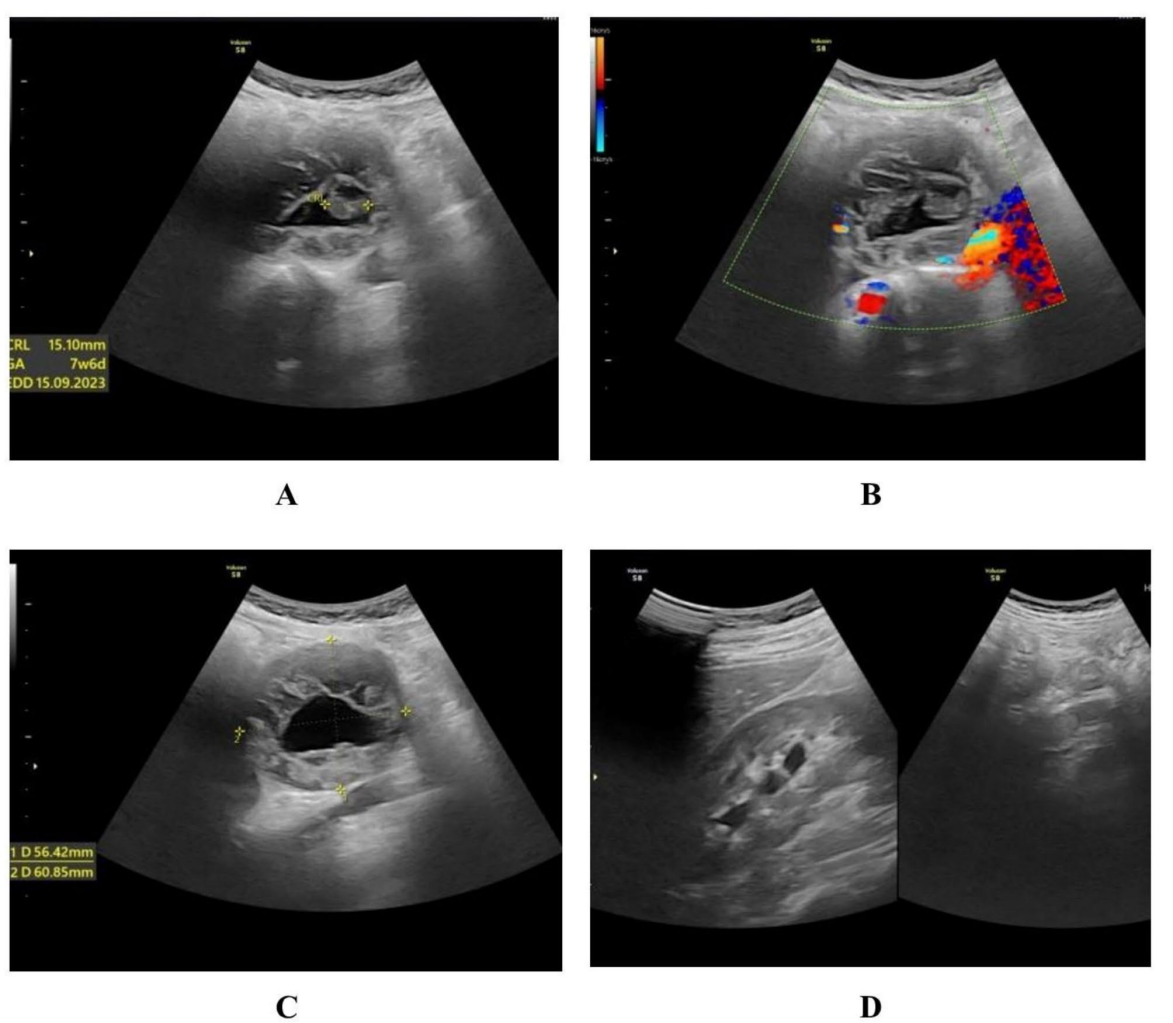
Transabdominal ultrasound in a reproductive-age woman (G5P2) revealed the following: a visible embryo 15 mm in length corresponding to 7 weeks and 6 days of gestational age without fetal heart activity (**A**). The mass is absent with a Doppler signal (**B**). The gestational sac measured 56 × 60 × 44 mm in dimension and was located in the right upper quadrant of the abdominal cavity (**C**). The kidneys and surrounding organs are normal (**D**)

**Fig. 2:**
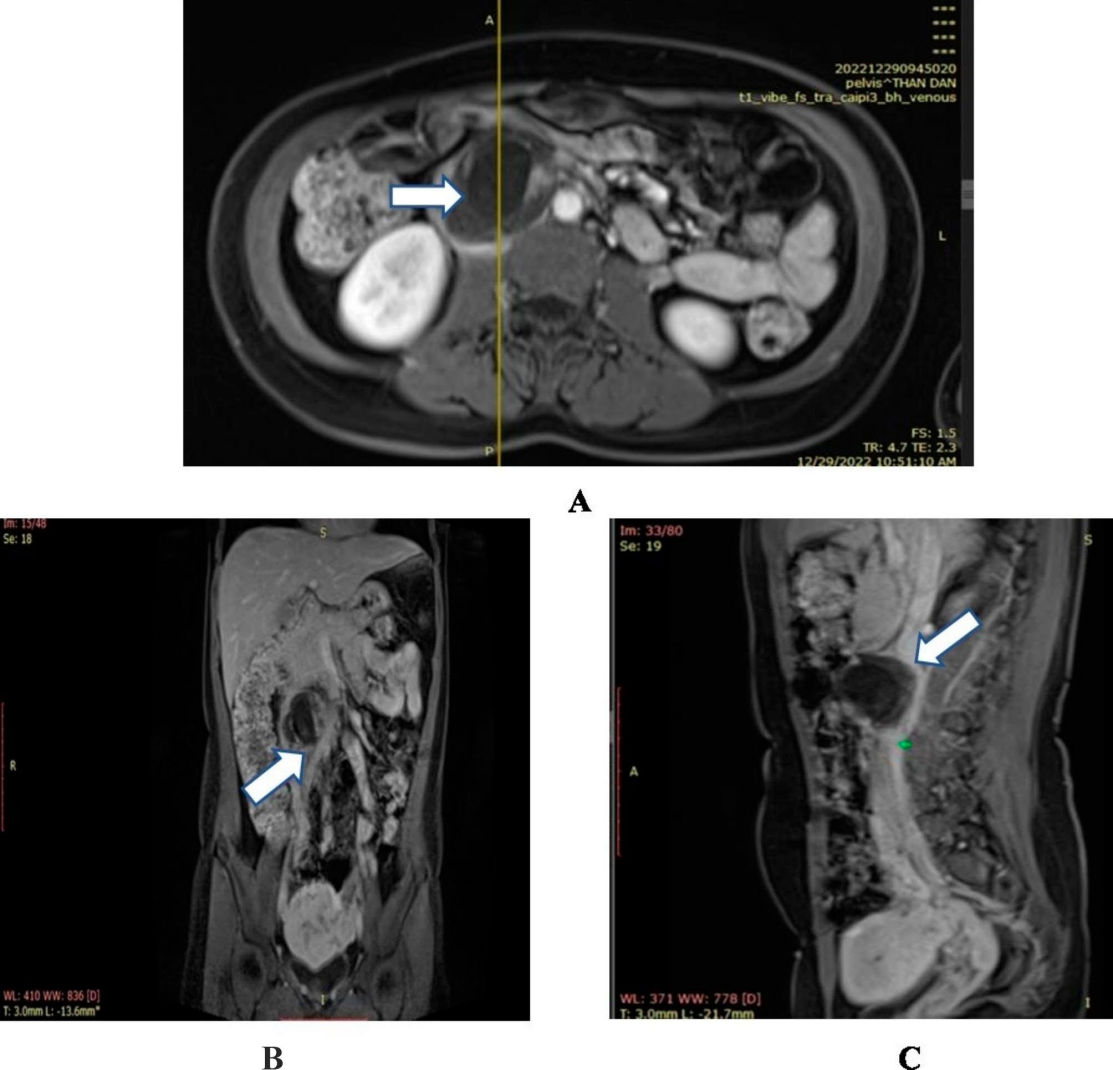
Magnetic resonance imaging in a 38-year-old female patient (G5P2) diagnosed a retroperitoneal ectopic pregnancy. Axial image showing a gestational sac measured 4.6cmx5.6cmx5.4 cm in size, invaded to the inferior vena cava in the retroperitoneal space, and tightly adherent to the abdominal aorta and duodenum (**A**). Coronal and sagittal images showed a gestational mass running from the lower pole of the right kidney, transverse to the vertebral column, and reaching to the bifurcation of the inferior mesenteric artery and two common iliac veins. The genital vein was dilated and in close contact with the gestational mass before attaching to the inferior vena cava. No fluid collection or hematoma was observed in the abdominal cavity (**B**-**C**).

Abdominal ultrasound showed no intrauterine gestational sac, with an endometrial thickness of 6 mm, and both adnexal structures were normal. Several fibroid nodules approximate 8–11 mm in diameter were detected within the intramural layer. After observing the lateral sides of the uterus and pelvis, the abdominal cavity was carefully scanned. An abnormal heterogeneous mass was found in the retroperitoneal cavity adjacent to the abdominal aorta, corresponding to a gestational sac at 7–9 weeks with a visible embryo, but absence of fetal cardiac activity (Fig. 1). Subsequently, MRI was performed to confirm the definite diagnosis and assist a deep investigation of the abdominal cavity (Fig. 2).

**Fig. 3:**
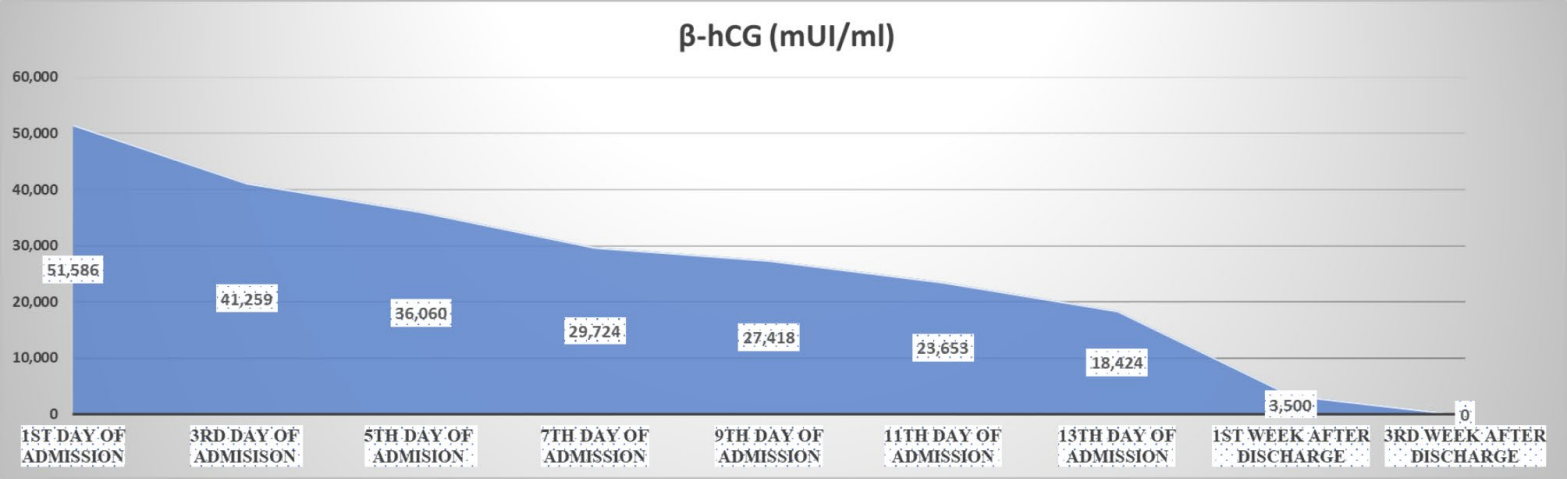
Variability of β-hCG levels during the expectant management of retroperitoneal ectopic pregnancy

Upon monitoring, the β-hCG concentration gradually decreased every 48 h (Fig. 3). After consultation, the expectant management was indicated. The patient was sent to home after 2-week hospitalization. The serum β-hCG level was monitored every week after discharge until negative value. Ultrasonography was repeated weekly along with β-hCG during the first 2 weeks after discharge. Then, ultrasound was followed-up monthly until the mass dramatically reduced in size without blood supply after six months. During outpatient follow-up, the patient was strictly monitored without any relevant complications.

Despite the lack of criteria for laparoscopic imaging and pathologic examination, the retroperitoneal mass reduced in size and the β-hCG level returned to the normal range after 1 month, and she continued to be stable after 3 months. This regression supported the confirmative diagnosis. The patient was thankful to the team for successful management without surgical or medical intervention.

## Discussion

In the present case, the patient had a retroperitoneal ectopic pregnancy with natural conception. This may be a rare occurrence since almost all cases have been reported relative to IVF treatment [16–18]. Retroperitoneal implantation of a fertilized ovum may be primary or secondary to a ruptured EP originating at another site. However, an original tendency of embryonic location alongside the great blood vessels and invasion into the lymph node tissue has been found in some cases [9, 18].

Accordingly, since the EP was hidden in the retroperitoneum, thus initial detection was relatively difficult. Therefore, it is necessary to rule out the possibility of EP and determine its location, especially alongside the large vessels [7]. Previously, Salma et al. reported a case of REP requiring 2 times of exploratory laparotomy since the diagnosis was neglected on the initial ultrasound and the first assessment of laparotomy [19]. Then, Park et al. also mentioned the similar case which its diagnosis was delayed on TAS and the first laparotomy until the presence of complication or elevated serum β-hCG levels after surgery [10]. In this case, since the presence of elevated serum β-hCG and no intrauterine pregnancy was observed on ultrasound, thus the team decided to perform the MRI scan to elucidate the ambiguous diagnosis. Although MRI is not the first tool in the assessment of REP in low-resource settings owing to its high cost, particularly, in the repeated indication of monitoring, it is often required following a suspected ultrasound to evaluate the surrounding vasculature [6, 18].

Generally, REP is a life-threatening condition because the gestational sac is located next to the fragile structures. Therefore, the trophoblastic tissue directly invades the neighboring organs. Consequently, it is very difficult to completely assess and remove REP mass because it is covered by the peritoneum. Hence, the placental invasion commonly results in adverse outcomes such as massive hemorrhage [10]. Owing to the limitations of the current data and insufficiently practical guidelines, the management of REP remains a challenge for physicians [1].

In the present case, the early pregnancy failure occurred spontaneously before hospitalization. The etiology of early fetal death may be caused by implantation in area unfavorable for fetal development. Therefore, the gestational sac size was naturally limited. In our patient, the β-hCG concentration gradually decreased. Furthermore, the clinical manifestations were absent with stable hemodynamic parameters. These advantages have contributed to the success of expectant management. The limitation of the non-surgical intervention was noted that our team could not assess the REP tissue for histopathological examination and exclude other malignant pathologies. However, since no sign of proliferative vascularity was detected on ultrasound, the mass size was gradually decreased, and the serum β-hCG levels returned to negative value. All these progressions supported for the benign pathology of ectopic pregnancy.

Similar to almost all ectopic pregnancies, treatment should be individualized and based on gestational age, gestational sac size, relative organs, clinical characteristics, β-hCG level, presence of fetal cardiac activity, and desire of the patient (Table 1). Although surgical intervention including laparoscopy and laparotomy is a rapidly effective therapy, it is highly related to severe complications and increased risk of vascular injury. Thus, a multidisciplinary team that includes a gynecologist, vascular surgeon, radiologist, and anesthetist is necessary in all cases [16, 17]. By summarizing of REP cases in the literature, Xu et al. concluded that multidisciplinary requirements substantially reduce surgical complications, thus increasing the survival rate of patients [7]. A methotrexate regimen can be used under suitable conditions to kill the trophoblast cells in the conservative management or reduce intraoperative hemorrhage [20]. A systemic administration of MTX in nonruptured REP before surgical method may be significant [21]. However, its side effects should be monitored and an adequate protocol is currently lacking [1].

## Conclusions

In summary, an importance of considering the possibility of REP should be emphasized in suspected cases of abdominal ectopic pregnancy with unknown location. In addition, expectant management can be considered in the case of nonviable pregnancy without complications, and multidisciplinary team could be immediately assessed.

## Data Availability

The datasets used and/or analyzed during the current study are available from the corresponding author upon reasonable request.

## List of abbreviations

β-hCG: beta-human chorionic gonadotropin
EP: ectopic preganncy
IVF: in vitro fertilization
MRI: magnetic resonance imaging
TAS: transabdominal ultrasonography
TVS: transvaginal sonography
REP: retroperitoneal ectopic pregnancy
